# Duplicate entries in the Protein Data Bank: how to detect and handle them

**DOI:** 10.1107/S2059798325001883

**Published:** 2025-03-08

**Authors:** Alexander Wlodawer, Zbigniew Dauter, Pawel Rubach, Wladek Minor, Mariusz Jaskolski, Ziqiu Jiang, William Jeffcott, Olga Anosova, Vitaliy Kurlin

**Affiliations:** a Center for Structural Biology, Center for Cancer Research National Cancer Institute Frederick MD21702 USA; b Department of Molecular Physiology and Biological Physics University of Virginia Charlottesville VA22908 USA; c Institute of Information Systems and Digital Economy Warsaw School of Economics Warsaw Poland; d Institute of Bioorganic Chemistry Polish Academy of Sciences Poznań Poland; e Department of Crystallography, Faculty of Chemistry, Adam Mickiewicz University, Poznań, Poland; f Department of Surgery and Cancer Imperial College London London United Kingdom; g Computer Science University of Liverpool LiverpoolL69 3BX United Kingdom; h Materials Innovation Factory University of Liverpool LiverpoolL69 3NY United Kingdom; University of Oxford, United Kingdom

**Keywords:** data duplication, Protein Data Bank, coordinate comparison, backbone rigid invariant

## Abstract

A global analysis of protein crystal structures in the Protein Data Bank (PDB) using a newly developed computational approach reveals many pairs with (nearly) identical main-chain coordinates. Such cases are identified and analyzed, showing that duplication is possible since the PDB does not currently have tools or mechanisms that would detect potentially duplicate submissions. Some duplicated entries represent modeling efforts of ligand binding that masquerade as experimentally determined structures. We propose that duplicate entries should either be obsoleted by the PDB or, as a minimum, marked with a clear ‘CAVEAT’ record that would alert potential users to the presence of such problems. We also suggest that using a tool for verifying the uniqueness of the deposited structure, such as that presented in this work, should become part of the routine validation procedure for new depositions.

## Abstract 2

A global analysis of protein crystal structures in the Protein Data Bank (PDB) reveals many pairs with (nearly) identical main-chain coordinates. Such cases are identified and analyzed, leading to a proposal about how the PDB could ameliorate this problem.

## 1. Introduction

**Figure 1:**
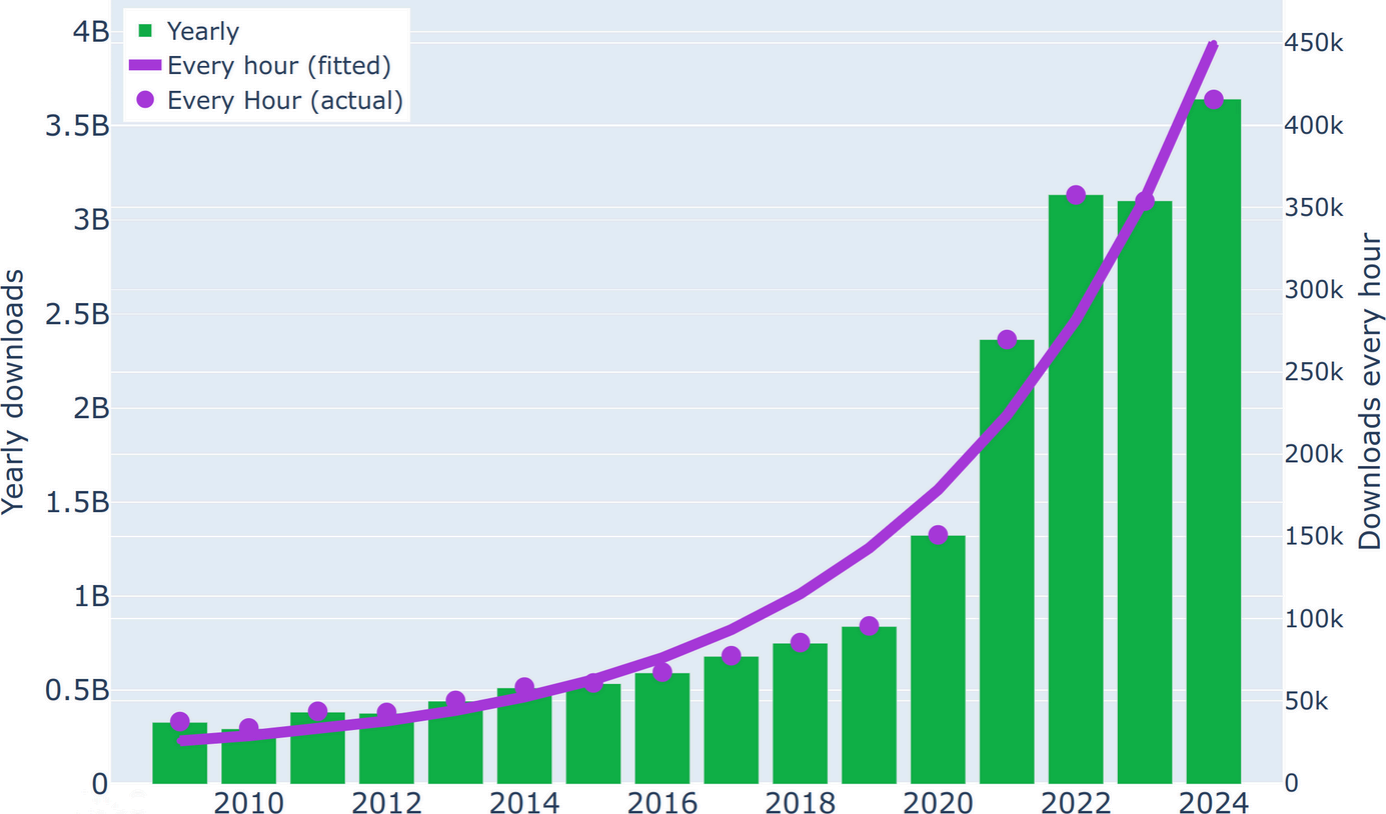
Total yearly and per-hour downloads from the PDB. Data from 2020 onwards include all types of experimental data, chemical reference data and validation reports obtained through HTTP, as well as coordinate files, structure-factor files and validation reports (via FTP only). The actual download numbers are represented by purple dots, while the purple line illustrates a fitted exponential function. The download statistics are from https://www.wwpdb.org/stats/download (Worldwide PDB, 2025 ▸). An interactive and updated version of this figure is available at https://bioreproducibility.org/figures/duplicate_entries/fig1/.

The Protein Data Bank (PDB; Burley *et al.*, 2018 ▸; Varadi *et al.*, 2022 ▸) is a treasure trove of structural biology that currently contains ∼230 000 macromolecular structures, and in this number there are over 190 000 crystal structures. Its importance to the scientific community cannot be overestimated, with millions of downloads every day (Fig. 1 ▸). Paradigm shifts leading to the development of structure-prediction tools, exemplified by *AlphaFold* (Jumper *et al.*, 2021 ▸), would not have been possible without using the contents of the PDB for the preparation of training sets. However, it must be emphasized that maintaining the highest possible data quality in this crucial repository is absolutely necessary. We have been campaigning for many years to eradicate different types of errors from the PDB, strongly believing that this venerated database will retain its high status only if we can detect and promptly eliminate the bad apples (Minor *et al.*, 2016 ▸) before they spoil the whole barrel. It has been indicated that such efforts should be the duty of the entire structural biology community (Wlodawer *et al.*, 2018 ▸), with a particularly responsible role befalling journal editors (Rupp *et al.*, 2016 ▸) and the PDB itself (Jaskolski *et al.*, 2022 ▸). It has also been pointed out that contamination of the PDB with accidental bad apples is especially dangerous for data mining (Dauter *et al.*, 2014 ▸), for the training of machine-learning algorithms and for rapid responses in situations of global biomedical threats (Grabowski *et al.*, 2021 ▸). So far, we have analyzed medicinally relevant segments of the PDB related to metal coordination and metallo-β-lactamases (Raczynska *et al.*, 2018 ▸), to complexes of cisplatin and carboplatin (Shabalin *et al.*, 2015 ▸), to SARS-CoV-2 proteins (Wlodawer *et al.*, 2020 ▸) and to l-asparaginases (Wlodawer, Dauter, Lubkowski *et al.*, 2024 ▸). In our most recent study, we have concentrated on the deposited coordinates themselves and discovered that many high-to-medium-resolution protein structures, including those of ultimately high resolution, are deposited in the PDB without any solvent molecules (Wlodawer, Dauter, Rubach *et al.*, 2024 ▸). Other authors have also sounded similar alerts, for example Kleywegt & Jones (1995 ▸), Kleywegt *et al.* (1996 ▸), Kleywegt (2000 ▸) and Armstrong *et al.* (2020 ▸).

With multiple entries available for a large number of proteins, the question of how to select the most representative ones has always niggled PDB users. This has recently been articulated, but not yet fully answered, in a note coauthored by a one-time head of the PDB (Bond & Sussman, 2024 ▸). However, even before this question can be addressed, it is important to make sure that the repository does not contain multiple models with identical or almost identical atomic parameters (coordinates and ADPs). The present work results from a collaboration between a team of crystallographers and a group of mathematicians who have developed efficient algorithms for the detection of (nearly) identical entries in large sets of numerical data and applied their tools to the detection of duplicate depositions in the PDB. The mathematical approach to this task has recently been described (Anosova *et al.*, 2025 ▸) and will be briefly summarized here.

To our surprise, the algorithm found many pairs of PDB protein models with (nearly) identical main-chain trace (N, C^α^, C atoms) coordinates, among which there are cases that truly have no rational explanation. This methodology, the results of its application, their analysis and proposals for remediation are the subject of the present paper.

## 2. Materials and methods

### 2.1. Outline of the mathematical approach to comparisons of tertiary structures

Most data objects, including protein structures, have infinitely many numerical representations (Anosova *et al.*, 2024 ▸). While all main-chain atoms in a protein backbone (N, C^α^, C, O) can be indexed uniquely, their coordinates are given in an arbitrary coordinate system. Any rigid transformation easily changes the atomic coordinates but keeps the underlying rigid shape, so that any coordinate representation is only one of infinitely many snapshots of a rigid object (Kurlin, 2024 ▸). Hence, coordinate comparisons cannot justify any conclusions, even in the case of two-dimensional lattices (Bright *et al.*, 2023 ▸). We note that both the ‘lock-and-key’ and ‘induced-fit’ models of protein interactions with ligand molecules motivate rigorous studies of continuous similarities between rigid shapes. Similarities traditionally based on the template-modeling (TM) score (Zhang & Skolnick, 2005 ▸) and the local distance difference test (LDDT) are known to fail the metric axioms (Mariani *et al.*, 2013 ▸), while the root-mean-square deviation (r.m.s.d.) test is slow for all-versus-all comparisons in the PDB (Holm, 2022 ▸).

Capitalizing on the previous successes in recognition of rigid shapes of infinite periodic structures (Widdowson & Kurlin, 2022 ▸) and molecules with indistinguishable atoms (Widdowson & Kurlin, 2023 ▸), the Data Science group at the Materials Innovation Factory (Liverpool, UK) developed a complete invariant of protein backbones (Anosova *et al.*, 2025 ▸). For a backbone of *m* residues, this invariant is a matrix of dimensions *m* × 9 describing the relative positions of the three backbone-trace atoms (N, C^α^, C) of each residue in a basis of vectors associated with the previous residue. The invariant can be uniquely inverted back to the backbone under rigid motion. Both the invariant and its inversion are continuous under perturbations, so that shifting any atom up to a small distance changes all invariant components to a constant multiple of this distance and vice versa. This backbone rigid invariant (BRI) distinguishes all mirror images and can be applied to any polymeric chain, not only protein backbones. More importantly, BRI is computed in a time that is linear in the number *m* of residues, while the classical distance matrix on 3*m* atoms has a quadratic size in *m* and cannot distinguish any mirror images in 3D. The crucial advantage of BRI is its simplification to the vector Brain (backbone rigid average invariant) of only nine coordinates by taking averages of nine columns in BRI. This averaging keeps the Brain invariant continuous. For torsion angles (in any fixed range, for example ±180°), their average for all residues is discontinuous at the endpoints because any angle close to +180° (such as +179.9°) can be perturbed to a value close to −180° (such as −179.9°). This discontinuity makes torsion angles insufficient for comparisons.

All-versus-all comparisons were performed using the approach described in Anosova *et al.* (2025 ▸). The supporting information contains the full definitions of invariants with examples.

### 2.2. Extraction and processing of protein data from the PDB

Extraction of data was completed using the PDB version of 4 May 2024 after filtering out 4513 nonproteins (entities labeled *not a protein*), 178 153 disordered chains in which some atoms have partial occupancies, 201 648 chains with residues having nonconsecutive numbers, 9941 incomplete chains missing one or more of the main-chain atoms and 4364 chains with nonstandard amino acids. If missing coordinates were only at the beginning or the end of a chain, these incomplete residues were removed and the shortened chain was retained in the cleaned data set. Future work will extend comparisons to more difficult cases, including inconsistent indices and disorder.

The remaining entries were separated into ∼707 000 individual chains. The first stage was to split all chains by the number *m* of residues, which is the simplest integer invariant. Even after this, the number of pairwise comparisons was more than 888 million. All comparisons were needed because anyone can take an existing protein chain from the PDB and replace many (or even all) amino-acid labels without changing the atomic coordinates or apply a rigid motion to all coordinates for extra disguise. Hence, comparing only the amino-acid sequences is insufficient. Similarly, comparisons of crystals by chemical composition only is unreliable, because artificial tools such as Google’s *GNoME* can easily replace atoms without changing their geometric positions and then report ‘2.2 million new crystals – equivalent to nearly 800 years’ worth of knowledge’ (https://deepmind.google/discover/blog/millions-of-new-materials-discovered-with-deep-learning); see Table 1 ▸ of Anosova *et al.* (2024 ▸).

The second stage was to filter out distant chains by comparing their average invariant Brain of only nine continuous coordinates. Indeed, if these average invariants differ by (say) 0.01 Å in at least one coordinate, the complete invariants can only have a larger difference in the same coordinate. Finally, for a much smaller subset of pairs of backbones with close Brain invariants, we use a fast nearest-neighbor search (Elkin & Kurlin, 2023 ▸) on the complete invariants BRI.

Comparisons of full chains already revealed thousands of exact duplicates. The invariant BRI(*S*) was designed to contain the invariant of any subchain of *S*. This property will facilitate a future search for (potentially many more) duplicate subchains by BRI in forthcoming work.

For a detailed inspection in this work, we limited the pool of structures to those with resolution 4 Å or better and rejected the entries labeled ‘Group deposition’ targeting the results of the *PanDDA* procedure, where multiple depositions have (by design) the same or very similar coordinates (Pearce *et al.*, 2017 ▸). The resulting data set consisted of 616 records that contained potentially duplicate structures (Supplementary File S1). The records that were used for actual comparison (Table 1 ▸) were checked again in December 2024, confirming that their status in the PDB had not changed since the original extraction.

## 3. Results

Automatic analysis of the contents of the PDB using the approach described in Section 2.1 led to the initial identification of 616 pairs of chains selected by the search criteria. Eliminating hits involving two parts of the same deposition decreased the number to 335 cases, and these structures were evaluated manually. We concluded that detailed analysis of structures of viruses and ribosomes would not be practical due to their complexity; thus, the final data set consisted of 56 pairs of depositions (Table 1 ▸), with at least three cases of more than two similar structures present.

### 3.1. Duplicate structures resulting from subsequent redeposition in the PDB

We found a number of entries in the PDB that were deposited several months to several years later than the initial deposition and that could be considered to be new versions; nevertheless, the original files were never obsoleted. In some cases both the metadata showing the details of data collection, as well as the results of structure refinement, are identical, with differences limited at most to some REMARK fields. Examples of such structures include Ile-tRNA synthetase (PDB entries 1ffy/1qu2; Silvian *et al.*, 1999 ▸), carbonic anhydrase IX (PDB entries 6oti/8fr1; Combs *et al.*, 2023 ▸) and programmed cell death protein 1 (PDB entries 8p1o/8r6q; Surmiak *et al.*, 2024 ▸). However, in most cases there are significant differences in the metadata, whereas all or almost all atomic coordinates and *B* factors are identical. The case of sucrose-specific porin (PDB entries 1a0t/1oh2; Forst *et al.*, 1998 ▸) has previously been identified as suspicious, since the most significant difference is the lack of solvent in the subsequent deposition (Wlodawer, Dauter, Rubach *et al.*, 2024 ▸), although the refinement statistics are identical. Strangely, the data-collection details seem to show significant differences (for example an *R*_sym_ of 0.155 versus an *R*_merge_ of 0.054). Although the authors were made aware of this problem more than two years ago, both depositions are still present in the PDB.

Results of a comparison of two depositions of the structure of the complex of the FK506-binding protein with human FRAP and rapamycin (PDB entries 1nsg/2fap; Liang *et al.*, 1999 ▸) indicate a situation that cannot be explained in crystallographic terms. Although different dates of data collection are listed in the PDB files, the statistics shown in the metadata are identical, despite a difference in the total number of reflections. Moreover, despite different unit-cell parameters, the atomic coordinates and *B* factors are exactly the same. Such an outcome is not possible if the structure was re-refined before being replaced. The differences in lattice parameters in the two structure-factor files that are otherwise identical cannot be explained by the application of any crystallo­graphically acceptable procedure.

The structure of tryptophan synthase complexed with glycerol phosphate was deposited as PDB entry 1wbj (Kulik *et al.*, 2005 ▸), whereas a complex with glyceraldehyde-3-phosphate was later deposited as PDB entry 2clk (Ngo *et al.*, 2007 ▸). Some differences between the data-collection metadata are present, but they do not indicate different structure factors. Indeed, the values of *F* and σ(*F*) are identical for each reflection. The only significant difference between these files is the nomenclature of the ligand, although the atomic coordinates and *B* factors of the G3P and G3H molecules are identical.

Quite significant differences in the data-collection metadata can be seen for the structure of FitAB bound to DNA (PDB entries 2bsq/2h1o; Mattison *et al.*, 2006 ▸), although the unit-cell parameters, atomic coordinates and *B* factors are identical in the two depositions. It seems that PDB entry 2h1o may have corrected some data-collection metadata present in PDB entry 2bsq, such as the impossible number of 16 708 unique reflections measured versus 33 243 used in a refinement that resulted in identical statistics. However, the free-*R*-factor flags present in PDB entry 2bsq were lost in PDB entry 2h1o.

The two depositions of the unpublished crystal structure of human P100 Tudor domain (PDB entries 2hqe/2o4x; N. Shaw, M. Zhao, C. Cheng, H. Xu, J. Yang, O. Silvennoinen, Z. Rao, B.-C. Wang & Z.-J. Liu, unpublished work) differ only by the addition of the OXT atom in the redeposition, without any change in all other atomic parameters. Additionally, the redeposition was accompanied by structure factors, whereas the original deposition was not.

The two depositions of the structure of KDPG aldolase (PDB entries 1wbh/2c0a; Fullerton *et al.*, 2006 ▸) report identical unit-cell parameters and refinement statistics, but there are significant differences in the data-collection metadata. Whereas the atomic coordinates and *B* factors are identical in the two models for the protein, some ANISOU records that are present for each atom in PDB entry 1wbh are not found in PDB entry 2c0a. The latter model includes several additional water molecules that must have been added without any subsequent refinement.

The originally deposited structure of phospholipase C (PDB entry 4f2u; Cheng *et al.*, 2012 ▸) was later updated (PDB entry 4i9m; Cheng *et al.*, 2013 ▸), although the original deposition was kept in the PDB. Whereas some data-collection metadata differ between these depositions, this is a clear case of duplication.

Structures of the ternary complex of human proteins CDK1, cyclin B and CKS2 bound to an inhibitor (PDB entries 4y72/5hq0; Brown *et al.*, 2015 ▸) are identical, although water molecules are numbered differently in the two models (while maintaining the same coordinates and *B* factors). Another similar case is represented by the structure of a complex of human RAS protein with Darpin K27 (PDB entries 5mlb/5o2s; Guillard *et al.*, 2017 ▸), in which the only difference between the two depositions is the date of data collection. These are clear cases of deposition duplication.

Although some data-collection details for the structure of the AsfvPolX–DNA5–dGTP ternary complex (PDB entries 5hrf/8ild; Qin *et al.*, 2023 ▸) exhibit substantial differences, the refinement statistics, unit-cell parameters and atomic parameters (including *B* factors) are identical. With redeposition performed years after the original deposition, we suspect that some data-collection details might not have been remembered and these two depositions should be considered as duplicates.

The two unpublished depositions of the structure of HLA complexed with a synthetic peptide (PDB entries 6vb4/6viu; R. J. Schutte, D. Li, J. Andring, R. McKenna & D. A. Ostrov, unpublished work) differ only by the absence of the OXT atom in the former model, while all other atoms have identical coordinates and *B* factors. Although the data-collection details vary, the structure-refinement statistics are the same. An unusual feature of both depositions is the lack of validation sliders that should accompany PDB entries on the rcsb.org webpage, although they are present on the PDBe webpage.

The atomic parameters of two grass carp interleukin-2 depositions (PDB entries 7cjn/7d9m; Wang *et al.*, 2021 ▸) are the same despite differences in the data-collection metadata. Data-collection dates differ for these very low-quality structures (both with *R*_free_ = 0.41), and the wavelength claimed for PDB entry 7cjn (0.97 Å) is impossible for data measured on a rotating-anode generator. The earlier model is an example of a rather careless approach to PDB deposition and is clearly redundant.

Different dates of data collection are also found in the depositions of the structure of influenza hemagglutinin (PDB entries 7wvd/8gv6; Chen *et al.*, 2022 ▸), with the only difference between the coordinate sets being the removal of three OXT atoms in the later deposition. Since all other coordinates and the refinement parameters are the same, this may be another example of modifying a model without subsequent refinement. The older deposition is clearly an unnecessary duplicate. Another structure described in the reference above concerns the antibody PN-SIA28, with two PDB depositions (PDB entries 7wvi/8gv4), again showing different metadata but identical refinement results. The number of protein and solvent atoms in the published manuscript does not agree with the number in either deposition, but it might be assumed that the later deposition should be kept and the older one removed.

Two depositions representing the structures of the complexes of GTP-binding protein Ran, Ran-specific GTPase-activating protein 1, exportin-1 and peptides Nm13 or Nm42 (PDB entries 6a3b/6kft; Sui *et al.*, 2021 ▸) are identical in all respects although they are supposed to contain different peptides. The statistics in PDB entry 6a3b agree with those in the publication for the complex with Nm13, but those in PDB entry 6kft, deposited a year later, do not agree with the statistics published for the Nm42 complex. The latter entry appears to be in error.

### 3.2. Structures deposited close together but practically identical

We noticed a number of cases where two or more structures deposited on the same date or within a few days are identical, or very similar. An example of a fully duplicated deposition is provided by the structure of mistletoe lectin I (inexplicably classified by the PDB as ribosome). Its two entries, 1ce7 and 2mll (Krauspenhaar *et al.*, 1999 ▸), deposited within two days of each other, are identical and only one of them should be retained. The published paper does not list the PDB code for the deposition, thus the choice of which one to retain is not obvious. Another clear case of a duplicated entry is provided by the structure of phosphoglucose isomerase (PDB entries 2pgi/1b0z; Sun *et al.*, 1999 ▸; Chou *et al.*, 2000 ▸), where the two files are identical with the exception of the *R*_free_ value (no structure factors were deposited with PDB entry 2pgi). The values of *R* and *R*_free_ in the validation report for PDB entry 1b0z do not agree with their counterparts in either deposition, most likely due to problems with *DCC* calculations, since they do match in the corresponding PDB_REDO entry.

The two entries for a *de novo* synthesized ATP-binding protein (PDB entries 3lt8/3lt9; Simmons *et al.*, 2010 ▸) are also identical, with the data-collection statistics in PDB entry 3lt9 agreeing with their counterparts in the publication for a complex formed with 100 m*M* ATP; yet the presence of 100 m*M* ATP is mentioned in the title of entry PDB entry 3lt8, confusing the issue further. The two structures of the sigmaAA domain 4 complex (PDB entries 4g94/4g6d; Osmundson *et al.*, 2012 ▸) also represent a clear case of duplication, with all relevant statistics being the same. The only difference between two entries for a complex of lyase with an inhibitor (PDB entries 6ebe/6eda; Nocentini *et al.*, 2018 ▸) is the date of data collection, otherwise these two entries are identical and only one should be retained. Two structures of bacterial chloride importer (PDB entries 6jy7/6jy9; Yun *et al.*, 2020 ▸) are also identical despite differences in the data-collection metadata. Another pair of structures from the same publication (PDB entries 6yk7/6mxc; Yun *et al.*, 2020 ▸) are identical in all respects other than the wavelength of the X-ray beam, and this is again an example of a clear duplication.

Two entries describing a 2.75 Å resolution crystal structure of the membrane type 1 matrix metalloproteinase with an inhibitor (PDB entries 1bqq/1buv; Fernandez-Catalan *et al.*, 1998 ▸) were deposited within a few days of each other. They are identical in all respects except for the presence of water molecules in PDB entry 1bqq but not in PDB entry 1buv (which was deposited later). As all reported refinement statistics are the same, they must be erroneous in at least one case, since the presence of 311 water molecules would certainly be reflected in the refinement statistics. Unfortunately, no structure factors were deposited in either case, so the question of which model corresponds to the claimed refinement statistics cannot be answered.

Although data-collection and refinement statistics differ in the two depositions of the complex of fructose-1,6-biphos­phatase with several ligands (PDB entries 1nv1/1nv5; Choe *et al.*, 2003 ▸), the unit-cell parameters and all atomic parameters (including *B* factors) are the same. This duplication might be the result of unintended deposition of the wrong file that was not detected by either the authors or the PDB. Since the details of data collection and refinement found in the PDB depositions do not exactly match those found in the publication (no PDB codes were reported), it is not possible to clarify what exactly happened and which entry represents the discussed structure. A similar situation is found for the depositions of the structure of the steroid receptor 2 DNA-binding domain in complex with a steroid response element (PDB entries 4oor/4ov7; Vetting *et al.*, 2015 ▸), where the unit-cell constants and all coordinates are the same, yet the details of data collection differ.

Two structures of inhibited trypsin (PDB entries 1o3g/1o3f; Katz *et al.*, 2003 ▸) were deposited simultaneously with a large number of other structures of several serine proteases. The details of data collection and refinement, as well as the unit-cell parameters, are different, yet the coordinates themselves are identical. The refinement *R* factors reported in the manuscript do not agree with those found in the PDB depositions, thus it is not possible to fully identify them. It must be emphasized that identical coordinates are incompatible with different unit-cell parameters; thus there is clearly a problem with these two depositions.

The refinement statistics and atomic coordinates (including *B* factors) for two depositions of an unpublished structure of neuraminidase (PDB entries 1w20/1w21; E. Rudino-Pinera, P. Tunnah, S. J. Crennell, R. G. Webster, W. G. Laver & E. F. Garman, unpublished work) are identical, except that four more water molecules are present in PDB entry 1w20. However, the data-collection statistics are not the same (with the data apparently collected on different days). The PDB entry 1w20 diffraction data are reported to have 2.15 Å resolution, yet the structure was refined at 2.08 Å. This case was reported to the authors two years ago, yet both depositions are still present in the PDB.

The two depositions of the crystal structure of a complex of the colicin E9 DNase domain with a mutant immunity protein IMME9 (PDB entries 2gzj/2gyk; P. S. Santi, O. O. Kolade, U. C. Kuhlmann, C. Kleanthous & A. M. Hemmings, unpublished work) differ in the date of data collection (with other details being the same), but are otherwise identical; thus, one of them should be obsoleted. A similar situation is found with two depositions of the structure of glucocorticoid receptor (PDB entries 3g9p/3g9o; Meijsing *et al.*, 2009 ▸), which differ in some data-collection statistics but are otherwise identical. Another clear case of duplication are two entries for cyclooxygenase-2 (PDB entries 4rrz/4rrw; Blobaum *et al.*, 2015 ▸), in which only the details of data collection are different. Some of the statistics in PDB entry 4rrw are wrong since the number of measured unique reflections is 28 734, whereas 91 293 were supposedly used for refinement.

The two depositions describing the crystal structure of the adenylate sensor from AMP-activated protein kinase (PDB entries 2qr1/2qrc; Jin *et al.*, 2007 ▸) most likely represent an effort to correct a deposition, although some puzzling aspects are present. Although some details of data collection are different, the refinement statistics are the same, other than the number of reflections used for this purpose. However, in PDB entry 2qr1 the *B* factors for residues Gly118-Gly119 are similar to those of the residues surrounding them, but in PDB entry 2qrc the *B* factors for Gly119 are 20.00 Å^2^, with the exception of O, and the coordinates are not the same. Thus it seems that PDB entry 2qr1 might be the correct entry and PDB entry 2qrc (deposited one day later) would represent an erroneous duplicate.

Two structures of ricin bound to antibodies V5G1 or V5G6 (PDB entries 7kc9/7kdm; Rudolph *et al.*, 2021 ▸) are identical, although the data-collection dates are different. Whereas the paper was supposed to provide crystallographic details in Table S1, no such table is present, and thus it is impossible to determine which of these two entries corresponds to the antibody referred to in the title.

The unit-cell parameters and atomic coordinates in two depositions of the complex of Gar transformylase with substrate and the inhibitors AGF302 or AGF305 (PDB entries 8fdz/8fe0; Tong *et al.*, 2023 ▸) are identical, with the exception that residue 905 was specified as Glu in PDB entry 8fdz and Ala in PDB entry 8fe0. Such a level of identity is very surprising in view of clearly different data-collection and refinement statistics. Since the unit-cell parameters listed in the publication agree with those of PDB entry 8fe0 (listed in the paper as 8ef0), it must be assumed that PDB entry 8fdz is incorrect. This conclusion is supported by the fact that the inhibitor present in both depositions is the same, despite differences in its identification in the PDB entry titles.

Although we have not analyzed ribosome structures in detail, we noticed, as an example, by a simple file comparison that the two entries describing the crystal structure of the 30S ribosomal subunit from *Thermus thermophilus* (PDB entries 4lf7/4lf8; H. Demirci, R. Belardinelli, J. Carr, F. Murphy IV, G. Jogl, A. E. Dahlberg & S. T. Gregory, unpublished work) are identical in all respects other than a few REMARK lines; thus they represent an unambiguous duplication.

### 3.3. Structures redeposited with some significant differences, but originals kept

Two structures of carboxypeptidase A complexed with very closely related inhibitors (PDB entries 1hdu/1hee; Cho *et al.*, 2002 ▸) provide an example of procedures that should never be followed. Although there are significant differences in the data-collection metadata (for example, the number of unique reflections at 1.75 Å resolution is 89 359 in PDB entry 1hdu and 105 084 in PDB entry 1hee), the two sets of coordinates are identical, differing only by the addition of four extra atoms in the ligands of PDB entry 1hee. The number of reflections reported in the refinement of each structure was 93 239 and the reported *R* factors are identical. The only conclusion that could be drawn from this case is that one of these entries represented a modeling effort in which extra atoms were added to the inhibitor that was otherwise identical to that present in the other entry. However, if this were the case the modeled structure should not be present in the PDB, since only experimentally derived and refined structures should be deposited there.

The PDB entries deposited for the structure of the FutA1 protein complexed with iron ions (PDB entries 2pt2/3f11; Koropatkin *et al.*, 2007 ▸) are certainly confusing. The only difference between these entries is the claimed oxidation state of the iron ion: ferrous [iron(II)] in PDB entry 2pt2 and ferric [iron(III)] in PDB entry 3f11, with the latter entry deposited over a year later. Whereas both depositions refer to the same publication, only PDB entry 2pt2 is listed there, where it is assumed that iron(II) is bound to the protein. No explanation for this change in interpretation is provided in the redeposited file, thus the presence of both depositions in the PDB must lead to significant confusion.

A comparison of two entries related to HIV-1 neutralizing antibody 2f5 provides an illustration of a reinterpretation and redeposition of a modified model without any attempt to re-refine it. There is no question that the diffraction data used in the refinement of PDB entry 2f5a (Bryson *et al.*, 2009 ▸) were the same as for PDB entry 2pr4 (Julien *et al.*, 2008 ▸), although the number of unique reflections was reported as 89 376 in the former deposition and 26 917 in the latter. The second number agrees with the 26 304 reflections claimed to be used for refinement in both cases. All atomic coordinates and *B* factors are identical in the two models, with the exception that the C-terminal carboxyl O atoms and residues 104–113 were removed from PDB entry 2pr4. This change is most likely to be responsible for the increase of the *R* factor from 0.235 to 0.240, since it was only computed without any re-refinement. This is a dubious procedure that should not be recommended, but at least in this case it is quite clear what the authors tried to accomplish. Nevertheless, the older deposition is redundant and should be obsoleted, or at least annotated as a duplicate with a CAVEAT record.

### 3.4. Other peculiarities found in the course of this analysis

Three depositions of staphylokinase (PDB entries 1c77, 1c78 and 1c79; Chen *et al.*, 2002 ▸) with two protomers in the asymmetric unit have identical coordinates for protomer *A* but utilize different symmetry-related protomers *B*. Temperature factors and occupancies for protomer *B* are present only in PDB entry 1c79, whereas they are set to zero in the other two depositions. The apparent purpose of this way of presenting the structures is to emphasize different possibilities for dimerization, with only dimer *A*–*A* supposedly corresponding to the dimer in solution. However, structure models without *B* factors clearly cannot be considered to be *experimental* and thus violate the PDB rule that only experimentally determined structures may be accepted.^1^ In addition, although only PDB entry 1c79 can be considered to be a complete entry, the model referred to in the publication (Chen *et al.*, 2002 ▸) is PDB entry 1c78.

Crystal structures of the HIV-1 protease complexed with two similar, but non-identical inhibitors (PDB entries 1npw/1npa; Smith *et al.*, 1997 ▸, 2003 ▸) have different unit-cell parameters, yet the atomic coordinates and *B* factors of the protein atoms and water molecules are exactly the same. Only the coordinates of the inhibitors differ between these two models. Since no details of the crystallographic procedures are listed in the respective publications, it is not possible to determine which of the two structures might represent the results of a true crystallographic refinement and which one was modeled based on the other one and would therefore be illegitimate as a PDB deposition.

The structures of two ligand complexes of krait venom phospholipase A_2_ (PDB entries 1po8/1tc8; Singh *et al.*, 2005 ▸) have some very peculiar properties. Whereas the unit-cell parameters and atomic coordinates of all protein atoms are exactly the same, the *B* factors are different. The coordinates and the *B* factors of water molecules show slight differences, and the two ligands are not identical. The refinement statistics are also not identical, although exactly the same number of reflections was used to refine both structures. A similar situation is found for two structures of trypsin–inhibitor complexes (PDB entries 1yyy/1zzz; Krishnan *et al.*, 1998 ▸). Although the unit-cell parameters show some differences, the atomic coordinates and *B* factors are identical for the protein, but slightly different for the water molecules. The ligands are different in the two depositions. These cases most likely are not due to deposition duplications, but rather due to doubtful nonstandard procedures during their determination.

The unit-cell parameters and the atomic coordinates are identical in two structures of aspartate transcarbamoylase (PDB entries 1rah/1rai; Kosman *et al.*, 1993 ▸), with the exception that residues 1–7 of protomer *D* are not present in PDB entry 1rai. However, the *B* factors differ between these two entries. There is not enough information to decide whether both structures were refined based on the same diffraction data; only minor differences between the refinement statistics are present. It is not clear whether these depositions represent duplication that resulted from some nonstandard refinement procedures or whether they represent separate experiments.

A very strange case of duplication is presented by the structures of type I ribosome-inactivating protein (PDB entries 4f9n/4i47; Kushwaha *et al.*, 2013 ▸). Whereas the diffraction data are clearly identical, the statistics of structure refinement exhibit small differences. Moreover, although the unit-cell parameters are also identical, the *X* coordinates of all atoms are 0.001 Å larger in PDB entry 4i47 than in PDB entry 4f9n, whereas the *Y* and *Z* coordinates and the *B* factors are identical. Another peculiar pair of structures, deposited by the same laboratory as in the case described above, correspond to bovine lactoferrin (PDB entries 5cry/5hbc; Rastogi *et al.*, 2016 ▸). Although the temperature of data collection is listed as different (300 K in PDB entry 5cry and 80 K in PDB entry 5hbc), all other data-collection parameters, as well as the structure-refinement statistics, are identical. In this case the *X* and *Z* coordinates are shifted by 0.008 and 0.002 Å, respectively, with the *B* factors being identical. We are unable to explain these results and it is not clear which models should be considered to be redundant/erroneous duplicates.

Two old structures of deoxyhemoglobin, PDB entries 3hhb (Fermi *et al.*, 1984 ▸) and 1gli (Vallone *et al.*, 1996 ▸), have almost identical unit-cell parameters and very similar coordinates, thus they were flagged in this comparison. However, the structures differ very significantly in their resolution (1.74 versus 2.5 Å) and clearly do not represent duplication. The latter data were collected on a single-counter diffractometer and it may be assumed that the unit-cell parameters were transferred from the previous crystal structure during data collection. This might have been a typical procedure at that time but is no longer relevant.

A series of structures of cytochrome *c* with ligands bound in a buried polar cavity were described in several publications from the same laboratory (Fitzgerald *et al.*, 1996 ▸; Musah *et al.*, 1997 ▸, 2002 ▸). Although the details of diffraction data collection differ for PDB entries 1aeu, 1aen and 1ac4, the unit-cell parameters are identical, as are all coordinates and *B* factors of all atoms, with the exception of the ligands. With the ligand *B* factors set to either 15.0 or 0.0 Å^2^, it can only be assumed that the ligands were simply grafted into a reference model and that these structures cannot be considered to be independently refined, despite the identical refinement statistics. Another series of these structures (PDB entries 1aeb, 1aed, 1aee, 1aef, 1aeg, 1aeh, 1aej, 1aek, 1aem, 1aeo and 1aeq) claims different unit-cell parameters to those of the structures mentioned above, but identical for the whole series. In this case, the coordinates are slightly different among the depositions, but the *B* factors of the inhibitors also indicate that they were never refined. No data-refinement statistics are present in these files and the unit-cell parameters do not correspond to those listed in the publication (Musah *et al.*, 2002 ▸). For these reasons, all of these depositions should be treated as models of inhibitor binding, but not as experimentally determined structures.

The depositions of two isomorphous structures of methane monooxygenase hydroxylase (PDB entries 1mty/1fzi; Whittington *et al.*, 2001 ▸) represent an example of the limits (and doubts) of an analysis that relies on finding almost strictly conserved elements in pairs of PDB crystal structures. Nevertheless, it illuminates other problems that need to be addressed. The older structure (PDB entry 1mty) was determined at a resolution of 1.7 Å and is mostly acceptable in geometrical terms, although structure factors were not deposited. The subsequently deposited PDB entry 1fzi model was obtained after subjecting the crystals to high-pressure xenon, but the resolution of the diffraction data is only 3.2 Å. It appears that the unit-cell parameters for the second structure were forced to be identical to those of the first one (which in itself is a dubious practice, since cell constants are correlated with thermodynamic conditions) and only very limited structure refinement was performed. For this reason, the protein coordinates of the xenon complex are almost the same as the original ones, with only the *B* factors being very different. Surprisingly, a large number of the *B* factors of PDB entry 1fzi are set exactly to zero, which is not an accepted procedure even for lower resolution structures and raises some serious questions about the validity of the refinement procedures used for PDB entry 1fzi.

## 4. Discussion

The PDB contains thousands of examples of X-ray crystal structures that have been redetermined for various legitimate reasons. One such reason is the advancement of crystallo­graphic methodology, and the numerous crystal structures of the classic hen egg-white lysozyme (∼1200 altogether) serve as the most pointed examples. Other reasons for such ‘duplications’ may be related to investigations of exciting drug-design targets, where numerous research groups contribute entries that together build a comprehensive picture of the research object. A good example here is HIV-1 protease, which from the time of its first structure elucidation in 1989 (Navia *et al.*, 1989 ▸; Wlodawer *et al.*, 1989 ▸) has become a major target of crystallographic studies, leading to a spectacularly successful (and also pathbreaking) structure-guided drug-design megaproject. It resulted in the approval of ten protease inhibitors by the FDA as AIDS drugs, of which several are still used in current clinical practice, saving the lives of AIDS patients.

Such multiple but independent protein crystal structure studies have the added advantage of providing multiple views of the protein structure in different chemical environments, helping to elucidate the potential energy landscape of the protein chain itself. This is somewhat akin to what is known in small-molecule crystallography as the principle of structural correlations or the structure-correlation method (SCM), first introduced by Hans-Beat Bürgi (Bürgi, 1973 ▸), where the behavior, or even reactivity, of small-molecule constituents can be gleaned from their observation in multiple crystallo­graphic contexts (‘fields’). Such correlations have led, for example, to the elucidation of the stereochemistry of nucleophilic attack on the carbonyl moiety (Bürgi *et al.*, 1973 ▸; Heathcock & Flippin, 1983 ▸) and to the mapping of many more stereochemical processes. It is very encouraging that the principle of SCM is nowadays finding its way into protein crystallography as well (attesting *inter alia* to the explosive growth of the PDB and to the continuous improvement of the quality of the macromolecular crystal structures therein), as exemplified by its recent applications to the stereochemistry of the asparaginase reaction (Lubkowski & Wlodawer, 2019 ▸; Pokrywka *et al.*, 2025 ▸).

However, the approaches described above are obviously not based on crystal structures that are exact duplicates of a given unique entry; on the contrary, they assume that no such duplications are used for this purpose. Indeed, any database of scientific data should be unique in the sense that it precludes the deposition of multiple copies of the same entry. This motto has been a silent assumption of not only the PDB but also the CSD (Cambridge Structural Database; Allen, 2002 ▸), which stores (now nearly 1.3 million) small-molecule crystal structures of organic and organometallic molecules.

With regard to the CSD, it was discovered in the past by Anthony Spek when testing his *PLATON* program (Spek, 2003 ▸, 2009 ▸, 2018 ▸) that almost identical entries had been redeposited with the same atomic coordinates, save for one atom which was substituted by another one (by the way, leading to some chemical inconsistencies that were overlooked by the perpetrators), with all of the fabrications ‘determined’ using the same experimental data set. The extreme example generated as many as 18 ‘original’ structures from one data set (Harrison *et al.*, 2010 ▸). Such malpractice, strongly suggestive of scientific fraud, could be at utmost leniency described as a complete ignorance of the principles of crystallography. In practical terms, they could mean a lack of proper supervision and responsibility or outright fabrication.

One would naively assume that such situations would not be found in the PDB, where the process leading to a macromolecular structure deposition is very time- and labor-intensive, with multiple checkpoints where things that might go awry could (and should) be detected and eliminated. Yet, real-life situations are quite different. Moreover (and quite surprisingly), the PDB does not have a mechanism for detecting and monitoring attempts to deposit an entry duplicating an existing structure. This is exactly what our analysis has revealed: that there are many pairs of depositions in the PDB where the coordinates of the protein main-chain trace atoms are the same or nearly the same. In many cases the similarity goes beyond the main-chain atoms and actually includes the whole structure. Occasionally, there might be one or a few atoms changed, or the ADPs might show evidence of manual manipulation.

There might be different explanations (but of course not justifications) behind such duplications, in addition to a lack of proper supervision and training, as mentioned above. For example, abandoned depositions might still get through, or different members of research groups might be acting without communication. Still, the users of the PDB would strongly hope that the PDB should be able to intercept and block such duplication attempts, or that at least it should alert the depositors of the existing issues. Whereas macromolecular coordinates and structure factors undergo extensive validation during the process of their deposition into the PDB, we are unaware of any checks that would prevent accidental (or intended) duplicate depositions. The software developed within this project might be a useful tool for implementation by the PDB for screening new deposition attempts.

Our comparison of each protein backbone in depositions available in the PDB to all other entries identified a significant number of cases of duplication. As we have shown in Section 3, these duplications fall into several categories. Obvious cases involved depositing the same structure twice when a number of structures were being deposited as part of the same project. In these cases the fault clearly lies with the depositors, but there is no clear mechanism regarding what to do where such duplication is discovered. The PDB can only obsolete entries with the agreement of the depositors, but if duplication is found years later even contacting them might not be straightforward. We also noticed that cases such as the PDB entry 1a0t/1oh2 pair, the authors of which acknowledged the duplication more than two years ago (see the appendix of Anosova *et al.*, 2025 ▸), have still not been remediated. We recommend that the duplicate entries be marked with an appropriate ‘CAVEAT’ record to make potential users aware of the problem.

A very annoying situation is represented by the cases of double or multiple structures that are supposed to represent independent refinement of the target protein with different ligands, yet in practice represent only a model grafted onto one of the experimental structures. These depositions clearly violate the policy of the PDB that allows only experimental structures to be deposited in the core database (the exception of Computed Structure Models mentioned in footnote 1 is quite a different story). The existing validation tools are not designed to detect such manipulation of structures, but such cases can easily be found with the method described here. It is our recommendation that such depositions should be, as a minimum, also marked with an unambiguous ‘CAVEAT’ record.

As has been our experience gained in the course of some earlier projects aimed at the analysis of the quality of PDB protein crystal structures (Wlodawer *et al.*, 2018 ▸; Jaskolski *et al.*, 2022 ▸; Wlodawer, Dauter, Lubkowski *et al.*, 2024 ▸; Wlodawer, Dauter, Rubach *et al.*, 2024 ▸), a detailed look at any group of depositions, selected by a particular property, always detects some problems unrelated to the original aim of the investigation. This was indeed the case here, where problems not related to the stated goal of finding duplicate entries have been also uncovered. Whereas some of these problems could be potentially detected and corrected by fully automated procedures (exemplified by *PDB_REDO*; Joosten *et al.*, 2012 ▸), some others could only be noticed by careful manual analysis of each structure.

The method developed and applied here is very efficient in identifying potentially duplicate entries in a large database such as the PDB. This cascade computation would be impossible for any distance metric, such as r.m.s.d., and is feasible only with the utilization of a hierarchy of invariants, from the simplest and fastest (*m* and Brain), to the complete BRI. All invariant computations and pairwise comparisons for ∼707 000 protein chains were completed within six hours on a modest desktop computer.

The Python code used for this purpose is available from the authors on request. We are also working on including the backbone rigid invariant and its distance metrics in the *CCP*4 software (Agirre *et al.*, 2023 ▸). We also suggest that our algorithm might be a useful addition to the PDB deposition toolkit, where it could work as an initial filter, checking whether a new deposition does not duplicate an existing entry.

We would like to emphasize once more that finding problems in some PDB entries should by no means be taken as criticism of the work of the international teams that curate this database. By using multiple validation methods, the PDB can make the depositors aware of potential problems, but their remediation is up to the authors of the structures. However, a more liberal and consistent marking of structures as potentially flawed might help the scientific community in selecting the best, noncontroversial and most representative structures in any research projects that rely on the knowledge of the three-dimensional structure of biological macromolecules.

## Supplementary Material

Definitions of the invariant and metrics with PDB examples. DOI: 10.1107/S2059798325001883/gm5112sup1.pdf

Supplementary File S1. Excel spreadsheet showing potentially duplicate entries. DOI: 10.1107/S2059798325001883/gm5112sup2.xlsx

## Figures and Tables

**Table 1:** Pairs of PDB depositions with (nearly) identical positions of protein main-chain atoms (N, C^α^, C) Resolution (Resol.) and maximum deviation between two corresponding atoms (Max. dev.) are given in Å. *R*_free_ is shown if reported in the PDB deposition. The number of residues that are identified as different in the two depositions is indicated as No. diff. res.

PDB ID 1	PDB ID 2	No. of residues	No. diff. res.	Resol. 1 (Å)	*R*_free_ 1	Resol. 2 (Å)	*R*_free_ 2	Max. dev. (Å)
1ac4 †	1aen	291	0	2.1	—	2.1	—	0
1aeb †	1aef	291	0	2.1	—	2.1	—	0
1buv	1bqq	184	0	2.75	0.248	2.75	0.248	0
1c77	1c78	130	0	2.3	0.267	2.3	0.267	0
1c79	1c78	130	0	2.3	0.267	2.3	0.267	0
1c79	1c77	130	0	2.3	0.267	2.3	0.267	0
1ffy	1qu2	917	0	2.2	0.281	2.2	0.281	0
1hdu	1hee	307	0	1.75	0.229	1.75	0.229	0
1n6j	1n6j	93	0	2.2	0.268	2.2	0.268	0
1npw	1npa	99	0	2.0	—	2.0	—	0
1nv5	1nv1	331	0	1.9	0.245	1.9	0.255	0
1o3g	1o3f	223	0	1.55	0.205	1.55	0.213	0
1oh2_*B*	1a0t_*B*	413	9	2.4	0.246	2.4	0.246	0
1oh2_*A*	1a0t_*A*	413	0	2.4	0.246	2.4	0.246	0
1rai	1rah	310	0	2.5	—	2.5	—	0
1tc8	1po8	118	0	2.7	0.233	2.71	0.2387	0
1w21	1w20	389	0	2.08	0.195	2.08	0.195	0
1wte	1wte	272	0	1.9	0.2232	1.9	0.2232	0
1yrq	1yrq	545	0	2.1	0.22	2.1	0.22	0
1zbl	1zbl	133	0	2.2	0.253	2.2	0.253	0
1zzz	1yyy	223	0	1.9	—	2.1	—	0
2bsq	2h1o	143	0	3.0	0.271	3.0	0.269	0
2c0a	1wbh	214	0	1.55	0.217	1.55	0.217	0
2clk	1wbj	390	0	1.5	0.202	1.5	0.202	0
2fap	1nsg	107	0	2.2	0.266	2.2	0.265	0
2gzj	2gyk	130	0	1.6	0.232	1.6	0.232	0
2mll_*A*	1ce7_*A*	241	1	2.7	0.319	2.7	0.319	0
2mll_*B*	1ce7_*B*	255	0	2.7	0.319	2.7	0.319	0
2o4x	2hqe	217	1	2.0	0.2492	2.0	0.2492	0
2pgi	1b0z	442	0	2.3	0.278	2.3	0.258	0
2pr4	2f5a	213	0	2.05	0.27	2.05	0.27	0
2pt2	3f11	316	0	2.0	0.212	2.0	0.212	0
2qr1	2qrc	91	0	2.7	0.2895	2.7	0.2895	0
3g9o	3g9p	75	0	1.65	0.2029	1.65	0.2029	0
3lt9	3lt8	69	0	2.55	0.239	2.55	0.239	0
4f2u	4i9m	304	0	2.19	0.2358	2.2	0.2358	0
4f9n	4i47	246	0	2.65	0.196	2.65	0.1993	0.001
4g94	4g6d	62	0	2.0	0.2426	2.0	0.2426	0
4lf8	4lf7	235	0	3.15	0.2054	3.15	0.2054	0
4ov7	4oor	73	0	2.7	0.2312	2.7	0.2326	0
4rrw	4rrz	552	0	2.57	0.2162	2.57	0.2162	0
4y72	5hq0	264	0	2.3	0.2519	2.3	0.2518	0
5cry	5hbc	348	0	2.79	0.27287	2.79	0.2727	0.003
5hrf	8ild	178	0	2.25	0.2632	2.25	0.2632	0
5mlb	5o2s	165	0	3.22	0.2354	3.22	0.2354	0
6eda	6ebe	257	0	1.88	0.2277	1.88	0.2277	0
6jy9	6jy7	264	0	1.9	0.1895	1.8	0.1895	0
6kft	6a3b	210	0	2.51	0.2544	2.51	0.2544	0
6viu	6vb4	99	0	2.33	0.2433	2.33	0.2433	0
6xk7	6xmc	362	0	1.85	0.2423	1.85	0.2423	0
7cjn	7d9m	115	0	2.66	0.4123	2.66	0.4117	0
7kc9	7kdm	258	0	2.3	0.2377	2.3	0.2377	0
8fdz	8fe0	200	1	2.48	0.2516	2.22	0.2516	0
8fr1	6oti	257	0	2.0	0.2543	2.0	0.2543	0
8gv6	7wvd	318	0	3.4	0.2614	3.39	0.2614	0
8p1o	8r6q	126	0	2.17	0.2894	2.17	0.2894	0

† These are representative pairs from among multiple depositions with identical unit-cell parameters within each group of PDB entries 1aeu, 1aen and 1ac4 and PDB entries 1aeb, 1aed, 1aee, 1aef, 1aeg, 1aeh, 1aej, 1aek, 1aem, 1aeo and 1aeq.

## Footnotes

1 This rule does not apply to *AlphaFold*-predicted structures, which form a separate category, Computed Structure Models (CSM).
