# Supplementary material for: Duplicate entries in the Protein Data Bank: how to detect and handle them
Source: Acta Crystallogr D Struct Biol. 2025 Mar 8;81(Pt 4):170–80. doi: 10.1107/S2059798325001883 (PMC11966240; doi:10.1107/S2059798325001883)
Supplement: Supplementary file 1 [file d-81-00170-sup1.pdf]

Acta Cryst D STRUCTURAL BIOLOGY

Volume 81 (2025)

Supporting information for article:

Duplicate entries in the Protein Data Bank: how to detect and handle them

Alexander Wlodawer, Zbigniew Dauter, Pawel Rubach, Wladek Minor, Mariusz Jaskolski, Ziqiu Jiang, William Jeffcott, Olga Anosova and Vitaliy Kurlin

1 Supplementary materials: definitions of the invariant and metrics with PDB examples

We start with the simpler *triangular invariant* that describes the rigid shape of each residue triangle $\triangle N_i A_i C_i$ on three main atoms per each of m residues: nitrogen N_i, α-carbon A_i, and carbonyl carbon C_i, for $i = 1, \dots, m$, see Fig. 1 (middle). For any points $A, B \in \mathbb{R}^3$, let $|\overrightarrow{AB}|$ be the Euclidean length of the vector $\overrightarrow{AB}$ from A to B. We denote vectors by $\boldsymbol{u} \in \mathbb{R}^3$, their *scalar* and *vector* products by $\boldsymbol{u} \cdot \boldsymbol{v}$ and $\boldsymbol{u} \times \boldsymbol{v}$, respectively.

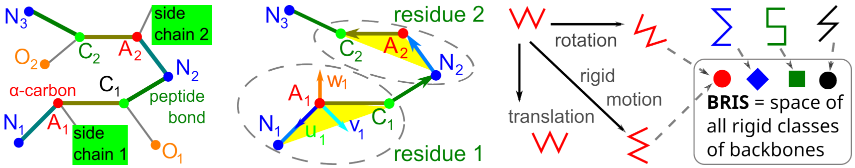

Figure 1. **Left**: all main atoms N_i, A_i, C_i of a protein chain form a *backbone* embedded in $\mathbb{R}^3$. **Middle**: each triangle $\triangle N_i A_i C_i$ defines an orthonormal basis $\boldsymbol{u}_i, \boldsymbol{v}_i, \boldsymbol{w}_i$. The coordinates of the bonds $\overrightarrow{C_i N_{i+1}}$, $\overrightarrow{N_{i+1} A_{i+1}}$, $\overrightarrow{N_{i+1} A_{i+1}}$ in this basis form the complete Backbone Rigid Invariant BRI. **Right**: All rigidly equivalent backbones form a single *rigid class*. All rigid classes of backbones form the *Backbone Rigid Space*.

Definition 1.1 (triangular invariant TRIN)**.** Let a backbone $S \subset \mathbb{R}^3$ have $3m$ ordered atoms N_i, A_i, C_i, $i = 1, \dots, m$. In the plane of $\triangle N_i A_i C_i$, for the 2D basis obtained by Gaussian orthogonalization of $\overrightarrow{A_i N_i}, \overrightarrow{A_i C_i}$, the vector $\overrightarrow{A_i N_i}$ has the coordinates $x(A_i N_i) = |\overrightarrow{A_i N_i}|$, $y(A_i N_i) = 0$, while $\overrightarrow{A_i C_i}$ has $x(A_i C_i) = \dfrac{\overrightarrow{A_i C_i} \cdot \overrightarrow{A_i N_i}}{|\overrightarrow{A_i N_i}|}$ and $y(A_i C_i) = \left| \overrightarrow{A_i C_i} - x(A_i C_i) \dfrac{\overrightarrow{A_i N_i}}{|\overrightarrow{A_i N_i}|} \right|$. The *triangular invariant* TRIN(S) is the $m \times 3$ matrix whose i-th row consists of the coordinates $x(A_i N_i), x(A_i C_i), y(A_i C_i)$ for $i = 1, \dots, m$.

The i-th row of TRIN(S) uniquely determines the shape of $\triangle N_i A_i C_i$. Many past approaches including AlphaFold2 assumed that all these residue triangles are rigidly equivalent. To test this assumption on the PDB, we filter out unsuitable chains as follows. On May 4, 2024, the PDB had 213,191 entries with 1,091,420 chains. Protocol 1.2 below produced $104,688 \approx 49\%$ entries with $707410 \approx 65\%$ chains in 4 hours 48 min 11 sec. All experiments were run on CPU Core i7-11700 @2.50GHz RAM 32Gb.

Protocol 1.2 (selecting a subset of 707K+ chains in the PDB)**.** The PDB was filtered by removing the following entries and individual chains.

(1) 4513 non-proteins (the entity is labeled as ‘not a protein’).
(2) 178153 disordered chains, where some atoms have occupancies < 1.
(3) 201648 chains with residues having non-consecutive indices.
(4) 9941 incomplete chains missing one of the main atoms N_i, A_i, C_i.
(5) 4364 chains with non-standard amino acids.

Example 1.3 (variability of residue triangles)**.** Fig. **??** (row 1) shows the heatmaps of the invariants $x(A_iN_i), x(A_iC_i), y(A_iC_i)$ on a logarithmic scale from Definition 1.1 across all 110+ million residues from the 707K+ cleaned backbones obtained by Protocol 1.2. Though standard deviations of these invariants are about 0.01Å, the maximum deviations of $x(A_iN_i), x(A_iC_i), y(A_iC_i)$ have high values of $1.2, 1.7, 2.7$Å, respectively.

Table 1 below shows the coordinates of TRIN and BRI (see Definition 1.4) for the two hemoglobin chains A in proteins 2hhb and 1hho, which are shown in Fig. 2 (top middle) and discussed in Example 1.6.

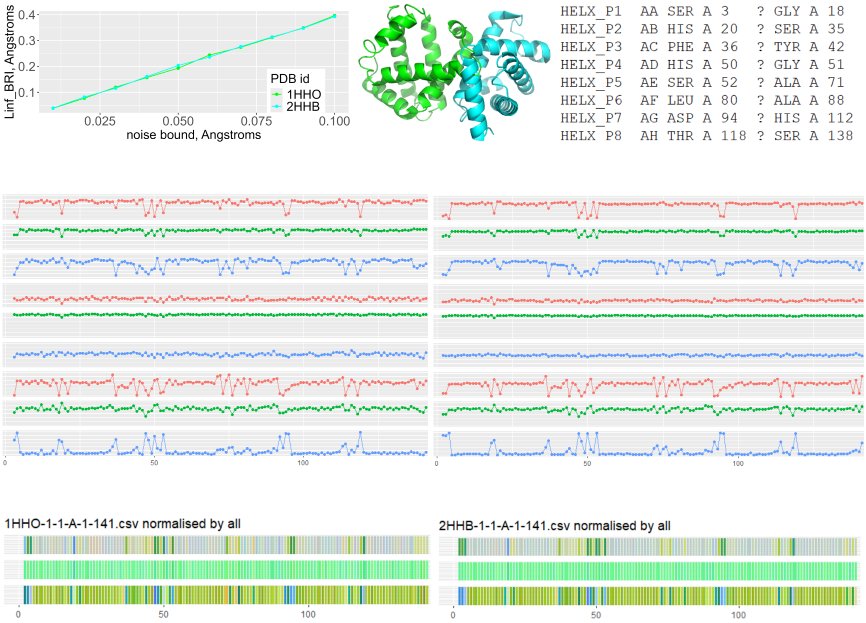

Figure 2. **Row 1**: the Lipschitz continuity of BRI is illustrated on the left by perturbing hemoglobins, where main chains A of 141 residues are shown in the middle (1hho in green, 2hhb in cyan) and eight α-helices extracted from the PDB on the right. **Row 2**: the Backbone Invariant Diagram (BID) of the hemoglobins 1hho vs 2hhb, see Definition 1.5. **Row 3**: the Backbone Invariant Barcode (BIB), see Example 1.6.

Table 1. Coordinates of TRIN and BRI for the first 3 residues of the chains A in 2hhb (top) and 1hho (bottom) with their means.

Res	$x(AN)$	$x(AC)$	$y(AC)$	$x(N)$	$y(N)$	$z(N)$	$x(A)$	$y(A)$	$z(A)$	$x(C)$	$y(C)$	$z(C)$
VAL	1.45	-0.54	1.44	1.45	0	0	0	0	0	-0.54	1.44	0
LEU	1.47	-0.50	1.47	-0.91	0.25	-0.90	-0.64	1.32	0.02	-1.10	0.01	1.10
SER	1.47	-0.48	1.45	-0.77	0.36	-0.98	-0.66	1.31	-0.05	-1.11	0.02	1.06
mean	1.47	-0.55	1.43	0.52	0.84	0.46	-0.48	1.38	0.05	0.01	0.65	-1.01

Res	$x(AN)$	$x(AC)$	$y(AC)$	$x(N)$	$y(N)$	$z(N)$	$x(A)$	$y(A)$	$z(A)$	$x(C)$	$y(C)$	$z(C)$
VAL	1.48	-0.51	1.46	1.48	0.00	0.00	0.00	0.00	0.00	-0.51	1.46	0.00
LEU	1.49	-0.55	1.42	-0.14	0.66	1.16	-0.69	1.31	0.19	-1.51	-0.16	-0.03
SER	1.44	-0.41	1.44	-0.63	0.27	-1.10	-0.36	1.36	-0.30	-1.43	0.14	0.40
mean	1.47	-0.53	1.43	0.56	0.81	0.44	-0.43	1.38	0.06	0.04	0.65	-1.02

Definition 1.4 will represent atoms $N_{i+1}, A_{i+1}, C_{i+1}$ in a basis of the previous i-th residue. The first residue needs only three invariants from Definition 1.1 to determine the rigid shape of $\triangle N_1 A_1 C_1$ in $\mathbb{R}^3$. Due to cleaning in Protocol 1.2, all consecutive atoms along any backbone have distances $d \geq 0.01$Å and all angles in any residue triangle $\triangle N_i A_i C_i$ are at least 3°, which makes the bases of all residue triangles well-defined.

Definition 1.4 (backbone rigid invariant BRI(S) of a protein backbone S)**.** In the notations of Definition 1.1, define the orthonormal basis vectors $\boldsymbol{u}_i = \dfrac{\overrightarrow{A_iN_i}}{|\overrightarrow{A_iN_i}|}$, $\boldsymbol{v}_i = \dfrac{\boldsymbol{h}_i}{|\boldsymbol{h}_i|}$ for $\boldsymbol{h}_i = \overrightarrow{A_iC_i} - b_i\overrightarrow{A_iN_i}$, $b_i = \dfrac{\overrightarrow{A_iC_i}\cdot\overrightarrow{A_iN_i}}{|\overrightarrow{A_iN_i}|^2}$, and $\boldsymbol{w}_i = \boldsymbol{u}_i \times \boldsymbol{v}_i$. The *backbone rigid invariant* BRI(S) is the $m \times 9$ matrix whose i-th row for $i = 2, \dots, m$ contains the coefficients x, y, z of the vectors $\overrightarrow{C_{i-1}N_i}$, $\overrightarrow{N_iA_i}$, $\overrightarrow{A_iC_i}$ in the basis $\boldsymbol{u}_{i-1}, \boldsymbol{v}_{i-1}, \boldsymbol{w}_{i-1}$. So the nine columns of BRI(S) contain the coordinates $x(N_i), y(N_i), z(N_i)$ of $\overrightarrow{C_{i-1}N_i}$, followed by the six coordinates $x(A_i), \dots, z(C_i)$. For $i = 1$, the first row of BRI(S) has only three non-zero coordinates $x(N_1) = x(A_1N_1)$, $x(C_1) = x(A_1C_1)$, $y(C_1) = y(A_1C_1)$ from the first row of TRIN(S) in Definition 1.1.

For a backbone of m residues, the first row of the $m \times 9$ matrix BRI(S) contains only three non-zero coordinates. Hence the matrix BRI(S) can be considered a vector of length $9(m-1)+3 = 9m-6$.

The simplest metric on backbone rigid invariants as vectors in $\mathbb{R}^{9m-6}$ is L_∞ equal to the maximum absolute difference between all corresponding coordinates. A small value δ of $L_\infty(\text{BRI}(S), \text{BRI}(Q))$ guarantees by that S, Q are closely matched by rigid motion, see all proofs in [1].

Definition 1.5 introduces the simplified (average) version of BRI, which allows a fast filtering of geometrically distant backbones.

Definition 1.5 (average invariant Brain, standard deviation of invariants, diagram BID, and barcode BIB)**.** For any protein backbone S of m residues, the *backbone rigid average invariant* $\text{Brain}(S) \in \mathbb{R}^9$ is the vector of nine column averages in $\text{BRI}(S)$ excluding the first row. The standard deviation can be computed in a similar way. The *backbone invariant diagram* $\text{BID}(S)$ consists of nine polygonal curves going through the points $(i, c(i))$, $i = 2, \dots, m$, where c is one of the coordinates (columns) of $\text{BRI}(S)$, see Fig. 2 (middle). For each atom type such as N, the coordinates $(x(N_i), y(N_i), z(N_i))$ are linearly converted into the RGB color value for $i = 1, \dots, m$. The resulting three color bars for the ordered atoms N, A, C form the *backbone invariant barcode* $\text{BIB}(S)$, see Fig. 2 (bottom).

Example 1.6 (hemoglobins)**.** The PDB contains thousands of hemoglobin structures. We consider here the structure 2hhb as a standard, and compare it with oxygenated 1hho, which contains an extra oxygen whose transport is facilitated by hemoglobin. In both cases, we considered the main chains (entity 1, model 1, chain A) of 141 residues. Table 1 showed the TRIN and BRI invariants for the first 3 residues of 2hhb and 1hho.

The top left image in Fig. 2 (top) shows that L_∞ on BRIs continuously changes under pertrubations for both hemoglobins. Fig. 2 (middle) illustrates the complexity of identifying similar proteins that can be given with very distant coordinates. The similarity under rigid motion becomes clearer by comparing their diagrams and barcodes in Fig. 2 (rows 2, 3).

More importantly, a rigidly repeated pattern such as α-helix or β-strand has constant invariants over several residue indices, which are easily detectable in BID and visible in BIB as intervals of uniform color.

The PDB uses the baseline algorithm DSSP (Define Secondary Structure of Proteins), which depends on several manual parameters and sometimes outputs α-helices of only two residues. For instance, the PDB files 1hho and 2hhb in Fig. 2 (right) include HELX_P4 consisting of only residues 50 and 51, and HELX_P5 of length 20 over residue indices $i = 52, \dots, 71$. Fig. 2 shows that a 'constant' interval of little noise appears only for $i = 54, \dots, 70$. Hence new invariants allow a more objective detection of secondary structures, which will be explored in future work.

References

[1] O. Anosova, A. Gorelov, W. Jeffcott, Z. Jiang, V. Kurlin, A complete and bi-continuous invariant of protein backbones under rigid motion, *MATCH Communications in Mathematical and in Computer Chemistry (to appear), arxiv:2410.08203* (2025).
